# Supplementary material for: Determination of IgG1 and IgG3 SARS-CoV-2 Spike Protein and Nucleocapsid Binding—Who Is Binding Who and Why?
Source: Int J Mol Sci. 2022 May 27;23(11):6050. doi: 10.3390/ijms23116050 (PMC9181569; doi:10.3390/ijms23116050)
Supplement: Supplementary file 1 [file ijms-23-06050-s001.zip › ijms-1665345-supplementary.pdf]

Cohort	n	Sex % male (M:F)	Median age yrs. (95%CI)	Ethnicity				
				white	mixed	Asian	black	other
**HCW-ve**	30	13% (4:26)	40 (36-45)	27	1	2	0	0
**HCW+ve**	32	25% (8:24)	44 (41-48)	26	1	4	1	0
**COVID-19**	37	72% (27:10)	52 (48-56)	31	1	4	0	1
		p< 0.001	*p<0.01*	*p=0.73*				

Co-morbidities COVID-19 cohort	
Hypertension	48.6%
Obesity (BMI>30)	35.1%
Type 2 Diabetes	29.7%
Respiratory Disease	8.1%
Heart Disease	8.1%

Figure s1 – Demographic details (sex, age and ethnicity) of the study cohort. HCW -ve are health care workers who were not antibody, antigen or PCR positive for SARS-Cov2 infection. HCW+ve are health care workers who were antibody, antigen or PCR positive for SARS-CoV2 infection. COVID-19 refers to patients confirmed positive for SARS-CoV2 and who had been admitted with acute respiratory distress syndrome (ARDS) to intensive care at Papworth Hospital, Cambridge, UK. The co-morbities sub table in the figure refers to the prevailing health conditions recorded for COVID-19 ARDS patient - cohort only.
